# Initial Afferent Lymphatic Vessels Controlling Outbound Leukocyte Traffic from Skin to Lymph Nodes

**DOI:** 10.3389/fimmu.2013.00433

**Published:** 2013-12-09

**Authors:** Alvaro Teijeira, Ana Rouzaut, Ignacio Melero

**Affiliations:** ^1^Centro de Investigación Médica Aplicada, Universidad de Navarra, Pamplona, Spain; ^2^Clínica Universitaria, Universidad de Navarra, Pamplona, Spain

**Keywords:** dendritic cell, T cell, lymphatic vessel, migration, inflammation

## Abstract

Tissue drains fluid and macromolecules through lymphatic vessels (LVs), which are lined by a specialized endothelium that expresses peculiar differentiation proteins, not found in blood vessels (i.e., LYVE-1, Podoplanin, PROX-1, and VEGFR-3). Lymphatic capillaries are characteristically devoid of a continuous basal membrane and are anchored to the ECM by elastic fibers that act as pulling ropes which open the vessel to avoid edema if tissue volume increases, as it occurs upon inflammation. LVs are also crucial for the transit of T lymphocytes and antigen presenting cells from tissue to draining lymph nodes (LN). Importantly, cell traffic control across lymphatic endothelium is differently regulated under resting and inflammatory conditions. Under steady-state non-inflammatory conditions, leukocytes enter into the lymphatic capillaries through basal membrane gaps (portals). This entrance is integrin-independent and seems to be mainly guided by CCL21 chemokine gradients acting on leukocytes expressing CCR7. In contrast, inflammatory processes in lymphatic capillaries involve a plethora of cytokines, chemokines, leukocyte integrins, and other adhesion molecules. Importantly, under inflammation a role for integrins and their ligands becomes apparent and, as a consequence, the number of leukocytes entering the lymphatic capillaries multiplies several-fold. Enhancing transmigration of dendritic cells en route to LN is conceivably useful for vaccination and cancer immunotherapy, whereas interference with such key mechanisms may ameliorate autoimmunity or excessive inflammation. Recent findings illustrate how, transient cell-to-cell interactions between lymphatic endothelial cells and leukocytes contribute to shape the subsequent behavior of leukocytes and condition the LV for subsequent trans-migratory events.

## Introduction

The lymphatic vascular system is composed by a one-direction system of conduits interrupted by lymph nodes (LN) that run in parallel to the blood vascular system.

Up to 50% of the protein that extravasates from blood vessels is reabsorbed by the lymphatic network (1). Besides, lymphatic vessels (LVs) in the gut are also devoted to the transport of absorbed lipids from the diet. One of the main functions of LVs is to conduit immune cells from tissues to the LN. This last function of the LVs constitutes the focus of this review. The cellular mechanisms and molecules involved in leukocyte transit across blood vessels have been studied in more detail and are generally assumed to be similar in the LVs (2). However, it must be stressed that although there are partial parallelisms in cell transit across these two types of vasculature, the diverse structure of the lymphatic capillaries may explain non-overlapping trans-migratory mechanisms. Even more, these peculiarities offer us new opportunities for selective therapeutic intervention to modulate leukocyte transit across the lymphatic capillaries.

The majority of the leukocyte populations that travels via the lymphatics to the LN are CD4^+^ lymphocytes, including effector memory and regulatory T cells (3–5). Myeloid cells also use the same conduits (6, 7), the majority of them being dendritic cells (DC) (8, 9). Myeloid cells are present in the lymph in lower amounts in homeostasis respect to T lymphocytes but their quantities significantly increase under inflammatory conditions.

Dendritic cells are key elements of the adaptive immunity that patrol peripheral tissues in search of pathogens or damage signals. Their main mission is to recognize and process foreign antigens in peripheral tissues and ferry them to the LN where they are presented to naïve T cells and then trigger an effective immune response.

In consequence, DC traffic toward LN via LVs has been extensively studied by immunologists in the recent years. Due to their abundance in the skin and its accessibility, DC transit in this tissue in homeostasis and inflammation has been the model used for most of the experimental studies. DCs from the skin include epidermal resident CD206^+^ Langerhans cells as well as dermal resident DC. Importantly, both cell types migrate at different times to the LN after contacting pathogen [for review Ref. (10)]. DC migration to the LN under steady-state conditions is constant and occurs at modest intensity (11) being instrumental to preserve peripheral tolerance to self-antigens (12–15). In contrast, under inflammation, DC migration toward the LN is significantly increased in response to chemotactic signals induced by inflammatory products (16). The amount of DC and specific subpopulation of antigen presenting DC (APC) entering into in the LN from peripheral tissues is important not only to elicit but also to sustain proper adaptive immune responses against pathogens. Therefore, the existence of regulation mechanisms for leukocyte-egress routes from the peripheral tissue is reasonable. Such mechanisms as consequence regulate leukocyte entrance into LN (16).

## Lymphatic Vessels Under Steady-State and Pro-Inflammatory Conditions

Leukocyte entry into the LVs is determined by the peculiar morphological features of these vessels: they are endowed with an intermittent basement membrane and their intercellular junctions are dispersed in button-like structures that leave small flaps of loose overlapping membrane extensions between individual LEC (17). Further, the LVs are attached to the extracellular matrix by anchoring fibers (18, 19) that stretch when tissue volume increases and lead to the opening the inter-endothelial flaps (19). It has been firmly established how under steady-state conditions DCs are able to penetrate into LVs via the preexisting pores (portals) of their basal membrane and subsequently migrate into LVs through inter-endothelial cell openings. This migration occurs in a process guided by chemokine gradients and mediated by contractions of the actin cytoskeleton, but is independent from integrin engagement, as it was shown in experiments performed in mice whose traceable DC are devoid of all integrins as bona fide pan-integrin knock-out DC mice (20).

It has recently been reported that during chronic inflammation or extensive lymphangiogenesis, there exists a transformation of the dispersed button-like adhesive structures of the mature lymphatic capillaries into ones more restrictive for cell transit featuring zipper-like contact adhesions similar to those present in collecting LVs and in blood capillaries. Importantly, this transformation is reversible and dependent on the activation of the glucocorticoid receptor by its phosphorylation (21). In fact, additional findings supported these observations. For example, it was reported how inflammatory cytokines (22) and pathogen-associated patterns (23) can promote VEGF-C production by the stromal cells and induce the formation of new LVs (lymphangiogenesis). In these models, inhibition of signaling across its receptor VEGFR-3 impaired the resolution of inflammation while its activation attenuated edema and induced the sprouting of new LVs (24–27). Besides, it has been extensively reported how increments in LVs facilitate the local resolution of the immune and inflammatory responses by augmenting DC transit across their boundaries (27, 28).

In line with this experimental evidence, it has been demonstrated how inflammatory mediators such as TNFα induce the up-regulation of integrin ligands on LVs surface such as VCAM and ICAM-1 and induce changes in the secretion of chemotactic cytokines both in *in vitro* and *in vivo* settings (29, 30). CCL21 is the main cytokine that drives DC migration to the LVs and its expression by LVs is strongly up-regulated upon exposure to pro-inflammatory cytokines such as TNFα (31). Nevertheless, other cytokines such as IFNγ limit LV proliferation (32) suggesting that transit of DCs across the lymphatic boundaries seems to be a phenomenon highly controlled by inflammatory mediators, although the precise molecules at work in each situation seem to vary. Indeed, previous reports from Vigl et al. showed how different models of inflammation (i.e., contact hypersensibility, CHS induction, or CFA injection) lead to diverse changes in LEC phenotypes in a stimulus-dependent manner (30). Other reports have demonstrated that the characteristic increment in transmural lymph flow that accompanies inflammation also results in greater CCL21 cytokine expression and leukocyte transmigration across LEC (33). Besides, to further support the role of this cytokine in DC migration Tal and co-workers have recently demonstrated by *in vivo* time-lapse microscopy that DC not only ingress the initial lymphatics through basal membrane deprived portals located in close proximity to CCL21 depots, but once inside the vessel these leukocytes crawl directionally on the luminal-side of the capillary. To crawl DC advance extending filopodia at their leading edges and retracting uropods formed at their rear end (34). These cells moved in a way that much resembles the inflammation-mediated integrin crawling of leukocytes inside the lumen of blood capillaries before their extravasation into tissues (35).

Peripheral inflammation is also able to promote effects in distant LN. Reports from Kinder and co-workers show how peripheral activated mast cells release micro particles that contain TNFα and other vasoactive mediators that facilitate leukocyte-egress toward the LN and induce the lymphangiogenesis of the lymphatic sinusoids in secondary lymphoid organs (36, 37). This new mechanism results in long distance-education of draining LN for the eventual reception of activated leukocytes (38, 39).

In addition to APC and effector T lymphocytes, T cells that infiltrate healthy or inflamed tissues may differentiate into a memory subset that expresses CCR7 and recirculates to LN (40–42). Similarly to DC, the arrival of antigen experienced T lymphocytes to LN is critical to regulate the intensity of immune responses. It is clear that there is much more detailed information regarding the mechanisms that drive the entrance of leukocytes to tissue to form inflammatory infiltrates than those that govern egress from tissue via afferent LVs. The most actively studied lymph-traveler leukocytes are DCs. However, memory T cell traffic for recirculation is also quite important and its understudied molecular mechanisms deserve much future attention, as is also the case with polymorphonuclear leukocytes (PMN).

Polymorphonuclear leukocytes upon acute inflammation are routed to LN via the lymph (43, 44) with potential to ferry antigens and pathogen-associated patterns (45) and regulate antigen presentation at the LN (43). Besides, DCs which have engulfed PMN also migrate via afferent LVs and may deliver PMN associated antigenic material to LN resident DC (45).

The experimental methodology used so far for the assessment of the interplay between leukocytes and LVs is quite similar to those in use for many years to study cell transit across blood capillaries and are being described next.

## Current Experimental Models to Study Leukocyte Transit Across Lymphatic Vessels

**Figure 1:**
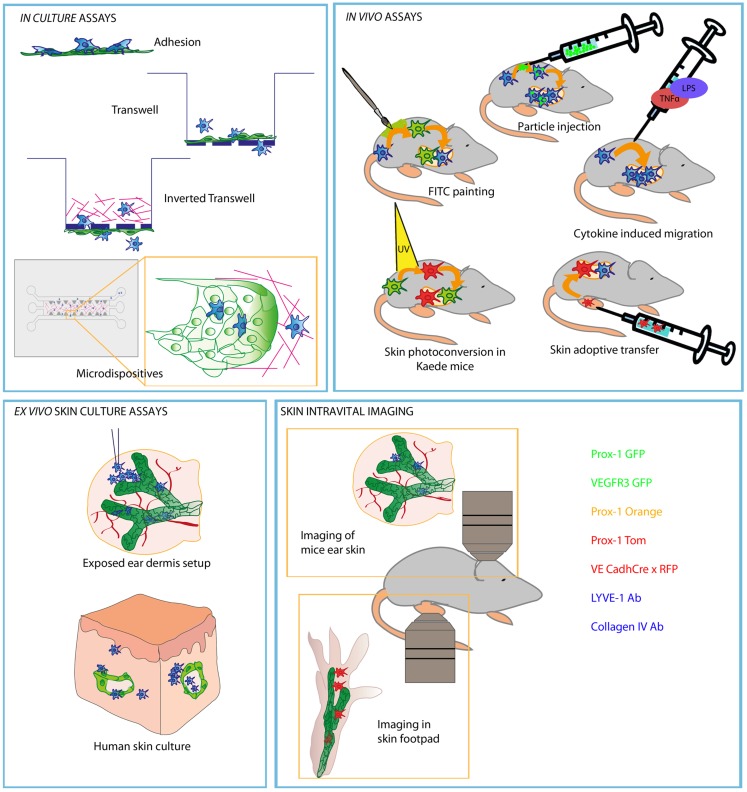
**Schematic picture representing most commonly used experiments to study leukocyte migration through afferent LVs**. Methods are divided in different compartments regarding the type of the experimental strategy and approach of each experiment. Lymphatic endothelial cells are represented in green and DC in blue (red or green when represented as stained).

Much of our current view on leukocyte transit across different lymphatic vascular beads depends on the experimental setting used. A graphical summary of such approaches is shown in Figure 1.

### Endothelial cell cultures

Human LECs can be purified based on the expression of specific markers (46) and are also commercially available as primary cells purified from human tissues such as dermis or lung. In this line, it is worth mentioning that LEC present different biological properties depending on their original tissue niche (47). From them, immortalized mouse LEC cell lines have been derived. The cell line most frequently used was purified from transgenic mice strains (SV-LEC) in which the expression of temperature sensitive SV40T antigen immortalized the lymphatic endothelial cells (48). This cell line is grown in special culture media and needs to be kept in culture at 33°C to maintain their immortalized status, making their use as routine experimental tool rather cumbersome. Other lymphatic endothelial cell lines derived from murine lymphoangiomas such as MELC cells have also been reported (49). This cell line retains most of the characteristics of primary lymphatic endothelial cells, but do not retain other features such as LYVE-1 expression. Still, they are a valuable source for research on lymphatic endothelial cell function since they can be *in vitro* propagated and assayed for transcriptional and functional assays. Primary isolated or immortalized lymphatic endothelial cells can also be transfected by liposome-based methodology and more powerful transfection techniques such as nucleofection or retrovirus mediated gene transfer (50, 51). Reliable methods to culture primary mouse LECs are not in place at the moment although it would offer definitive advantages.

The *in vitro* study of leukocyte adhesion to LEC monolayers provides quantitative data and permits experiments with blocking monoclonal antibodies to assess the role of different adhesion molecules (29, 52, 53) as a preliminary tool to describe cell transit across these vessels. Besides, this set up allows DC imaging on fixed and live specimens of lymphatic endothelial cells (33, 54, 55). In contrast, one of the mayor limitations of this experimental approach derives from the fact that leukocyte binding to monolayers of LEC adhered to plastic or ECM coated wells ignores the apical to basal polarization of the endothelial cells. Even though a lack of polarization has been reported for these cells *in culture* (29), in those experiments, DC used the same molecules to adhere to either the apical or the basal face of the endothelium (56). However, more research is needed about the importance of polarity for cell transit across LVs.

A second feasible and reproducible *in vitro* assay relies on the use of transwell systems in which the endothelial cells are seeded on the bottom of the transmigration filter between chambers. Migratory cells are then added on the upper well of the camber and allowed to cross the endothelium following a chemotactic gradient originated in the bottom well of the dispositive. This methodology also provides the researcher with a quantitative system to assess chemokine-driven active migration across monolayers. By using blocking monoclonal antibodies and decoy receptors the contribution of each of the different receptors can also be tested. An advantage of the use of transwell assays is the possibility to analyze basal to apical migration, but attention should be paid to pore size and the type of ECM proteins used to coat the transmigration membranes between chambers (33, 54).

Novel approaches in cell culture have been developed to grow endothelial structures in 3D micro-devices that allow both physicochemical and confocal microscope-based analysis of cell transmigration across endothelial tubules (57–59). These devices have been mainly applied to study tumor cell biology and metastasis but are promising in the context of cell migration across LEC as an attractive system for live imaging of leukocyte-LEC interaction.

### *Ex vivo* assays

This experimental approach is based on the isolation and culture *in vitro* of skin samples obtained from animals or surgery samples, under sterile conditions. It has been used for the study of DCs emigration from tissue (60). This technical approach simulates more accurately the physiological context than the previously mentioned *in vitro* assays. In this *ex vivo* set up, the visualization of tissue-resident DC in their way across LVs by confocal microscopy is challenging due to the scarce number of endogenous DC in the tissue sample and their lack of motility once explanted from tissue. Therefore, it is more feasible to directly add *in vitro* differentiated DC onto the explanted skin samples or inject traceable DC to the animals before sacrifice. Using this methodology leukocyte migration toward LVs has been observed by *in situ* imaging (61) and quantitative assays in this respect have been reported (29, 62). Though offering evident advantages, tissue explants also present some drawbacks because: (i) DC may abandon the tissue using other routes different from LVs; (ii) explanted tissue offers limited viability in culture (3 days, the longest in our hands); (iii) the experiments are performed in the absence of lymph flow.

### *In vivo* models

The main *in vivo* approximations for the study of DC migration across LVs have been reviewed in depth elsewhere (63). Briefly, current functional assays for the study of leukocyte migration toward the LNs are based largely in two different experimental approaches: the application of different inflammatory stimuli to induce endogenous DC migration, and the adoptive transfer of *in vitro* differentiated DC. The first type of experiments include FITC painting (16, 64), injection of fluorochrome-loaded particles (65), or the application of pro-inflammatory agents to the skin (66). FITC painting allows easy tracing of those leukocytes that have migrated out from skin since they are fluorescent. A pitfall of this methodology is that FITC molecule works as hapten and produces inflammation *per se*, hence being unsuitable for the investigation of steady-state cell migration. Injection of labeled microparticles is used to follow phagocytic DC populations and presents the advantage of allowing the study of DC migration under steady-state conditions. In addition, since these microparticles can be labeled with different fluorochromes, multiple DC populations can be traced in one single experiment. Direct injection or application of pro-inflammatory agents on the skin makes possible the study of the changes in endogenous DC populations both in the skin and in the draining LN. This approximation is useful to assess differential immunological responses to diverse inflammatory stimuli. However, with this technique it is not possible to directly follow DC migratory events. To overcome this problem, mice engineered to express the photoconvertible fluorescence protein Kaede, which changes from green to red when exposed to violet light have been used in experiments to trace the destiny of different leukocyte populations. In this line, Tomura and co-workers trace skin lymphocytes routed to draining LNs after skin exposure to violet light (5). Again, it is likely that photoconversion might act as an inflammatory stimulus.

More accessible methods are based on the adoptive transfer of pre-stained or genetically tagged DCs or T cells. This approach facilitates the study of the migration of leukocytes obtained from different transgenic models, while keeping a wild type background in the receptor mice (53, 67). The main limitations of this technique derive from the fact that experiments are usually performed with DC differentiated in culture and not with actual tissue-resident populations. It should be noted that DC are phenotypically diverse and may respond differently to environmental stimuli. Besides, the injection of the cells directly into the tissue may produce minor inflammation and undesired leukocyte activation.

Recently, intravital imaging has become a powerful tool to dissect the biology of leukocyte intravasation into the LVs. This approach captures images of cells as they migrate toward and traverse the lymphatic endothelium in live animals in a non-invasive fashion. For this purpose, increasingly sophisticated two-photon fluorescent microscopy technology leads our progress. Indeed, this methodology has provided crucial *in vivo* information about DC migration across LVs in steady-state conditions (34, 55). Besides, many mouse models have been developed to visualize LVs by *in vivo* microscopy based on the selective expression of fluorescent proteins under the regulation of different promoters (55, 68–72) or by the use of fluorescence-labeled antibodies (34, 73). Non-invasiveness and performance of the experiments in intact animals allows continuous lymph flow and makes this approach very suitable, since the main shortcomings from the previously explained methodologies are overcome. Yet, there are limitations to *in vivo* microscopy such as paucity of relevant transgenic animals expressing fluorescent protein under suitable promoters to study leukocyte entrance into lymphatics, and the fact that experimentation with human cells in this setting is impossible.

## Adhesion Molecules, Cytokines, and Chemokines that Control Leukocyte Transit into Lymphatic Vessels

### Chemokines involved in leukocyte trafficking into LVs

Dendritic cells and T cells follow chemotactic gradients that lead them toward the LVs and facilitate their transmigration and crawl on the luminal-side of the lymphatic capillaries until they reach the wider collector vessels from where lymph flow drifts them toward LNs (34). The role of the chemokine receptors and adhesive molecules and the models in which they have been investigated are summarized in Table 1.

**Table 1:** **Summarizing table of the main molecular players described in Leukocyte traffic through LVs**.

LEC receptor/ligand	Leukocyte receptor/ligand	Experimental model	Key observations	Reference
CCL21	CCR7	DC and T adoptive transfer of CCR7^−/−^, LN analysis on CCR7^−/−^mice, IVM of footpad	DC, lymphocytes, and neutrophils fail to migrate into LVs to LNs both under steady-state and inflammatory conditions	(11, 34, 40, 41, 67, 78)
CXCL12	CXCR4	FITC painting in the presence of a chemical inhibitor, transwell assays, inflammation models	Impaired DC migration to LN when treated with inhibitor. Induces transmigration across LEC in transwell assays. Impaired CHS response when inhibited	(62, 85)
S1P	S1PR1-5	FITC painting in the presence of chemical inhibitor FTY720, DC analysis in LN under inflammation and in the presence of inhibitor, adoptive skin transfer of T lymphocytes, and whole mount immunofluorescence after ear injection in FTY720 treated cells	Impaired DC and T cell migration from skin to LNs. Induced by inhibitor. Impaired *in vitro* trans-endothelial migration in LVs. Induces transmigration across LEC in transwell assays	(54, 91, 92, 97)
D6	Inflammatory chemokines	Immunofluorescence of skin LVs and LNs and study of LN DC populations after TPA induced inflammation in D6^−/−^mice	Accumulated inflammatory cells blocking LV function and other DC migration in D6^−/−^mice	(105)
CX3CL1	CX3CR1	FITC painting in CHS preinflamed skin in the presence of CX3CL1 Abs, adoptive transfer of CX3CL1^−/−^BMDCs, transwell assays	DC migration to LN is delayed. Impaired *in vitro* migration when the chemokine or its secretion is blocked. Effects only observed under inflammation	(99)
PECAM	PECAM	Transwell assays, *ex vivo* human skin culture in the presence of blocking Ab, immunofluorescence, and DC count inside LVs	Impaired trans-endothelial migration and intravasation in human skin explants, evidence provided only in human	(62)
ICAM-1	CD11a,b	FITC painting in the presence of blocking antibodies or in ICAM-1 deficient mouse, BMDC adoptive transfer in the presence of blocking Abs or from CD18^−/−^mice, whole mount immunofluorescence of ears after DC injection, transwell assays	In inflammatory and high flow conditions Blockade of ICAM-1 and blockade or β2 integrins inhibit trans-endothelial migration and DC migration to LN	(29, 33, 56, 107, 108)
VCAM	VLA-4	BMDC adoptive transfer in the presence of blocking Abs, transwell assays	LN impaired DC migration under inflammation and impaired *in vitro* trans-endothelial migration	(29)
CD99	PILR	Transwell assays, *ex vivo* human skin culture in the presence of blocking Ab, immunofluorescence, and DC count inside LVs	Impaired trans-endothelial migration and intravasation in human skin explants, evidence provided only in human	(62)
L1CAM	L1CAM	FITC painting assays in mice deficient in L1CAM under Tie 2 promoter, transwell, and adhesion assays	Impaired adhesion, transmigration in human (moDC) and mice (BMDC), impaired migration to LN	(52)
ALCAM	CD6	Lung injection of FITC microbeads in ALCAM^−/−^mice	Impaired DC arrival to LNs in ALCAM^−/−^mice	(119)
Podoplanin	CLEC-2	FITC painting in CLEC1b^−/−^mice, adoptive transfer of CLEC1b^−/−^DC, immunofluorescence of mice ear dermis cultured with BMDC	Impaired arrival of DC to LN, impaired intravasation, importance for protrusion formation	(123)
JAM-A	JAM-A	CHS response and FITC painting in Jam-A^−/−^and adoptive transfer of Jam-A deficient BM DCs, transwell assays	Jam-A ablation increases DC migration to LN and CHS responses as well as *in vitro* TEM	(161)
CLEVER-1	–	Adoptive lymphocyte transfer in the presence of blocking Abs in mice and rabbit	Lymph node migration of lymphocytes is blocked	(127)
Mannose receptor	Glycoproteins	Adoptive lymphocyte transfer in footpad of MR^−/−^mice and IF of LNs	Lymphocyte migration to LN and adhesion to LEC in LNs is impaired	(130)
Semaphorin 3A	Plexin-A1	Adoptive transfer of Plexin-A1^−/−^BMDCs, or wt DCs in Sema3A^−/−^mice, functional studies of T cell responses upon OVA skin sensitization in Plexin-A1^−/−^mice. *In vitro* videomicroscopy	Both, Plexin-A1 and Sema3A absence impairs DC migration to LNs. Sema3A is able to induce actomyosin contraction in BMDCs	(125)

*The table includes ligands expressed on both leukocyte and LEC, the evidence provided in leukocyte traffic and the experimental approach performed*.

The main chemokine-chemokine receptor system that controls leukocyte migration to LNs is CCL21/CCL19-CCR7 axis. The CCR7 chemokine receptor is expressed on DC under steady-state conditions but is strongly up-regulated upon maturation (74). This chemokine receptor has been described as instrumental for DC migration to LNs in experiments using adoptively transferred or autochthonous dermal DCs both under inflammation and steady-state conditions (11, 67). The same function applies for T lymphocyte (40, 41) and neutrophil (44) migration into the LVs.

**Figure 2:**
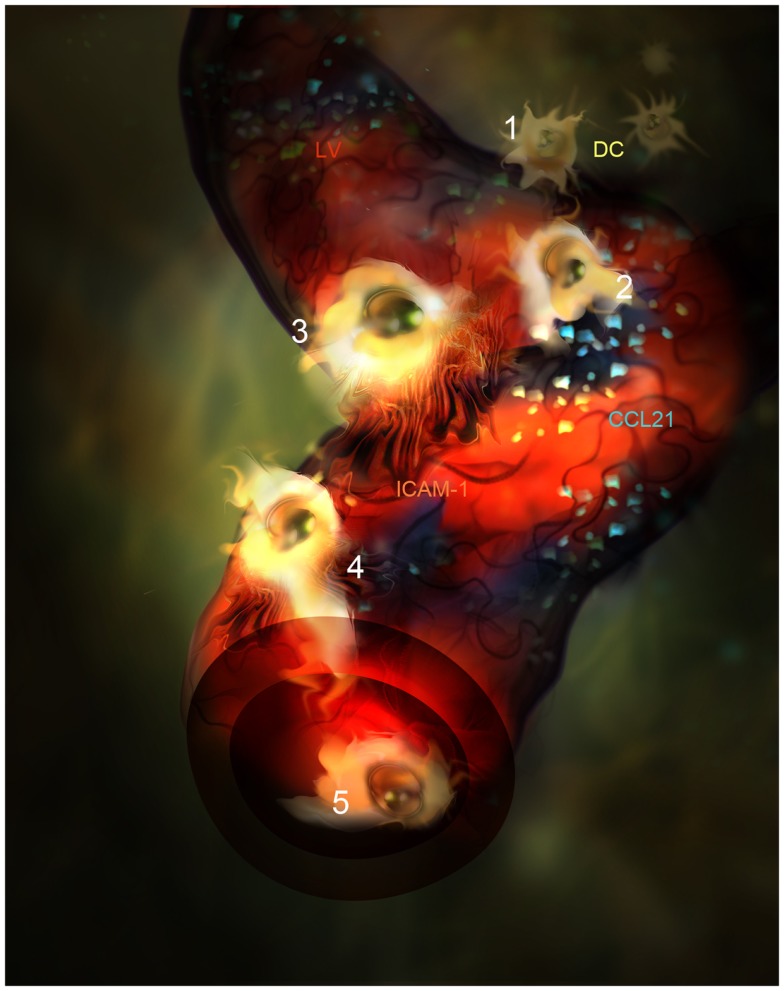
**Artistic representation of novel steps in DC migration into LVs under inflammatory conditions**. (1) DC (Yellow) are attracted by CCL21 interstitial gradient and other chemokines toward LVs lined by specialized endothelial cells (red). (2) DCs dock to CCL21 (blue) adsorbed as enriched patches prior to adhesion and intravasation. (3) DCs adhere to LEC surface and get entrapped by ICAM-1 enriched microvilli projections(Orange). (4) DC intravasate in a LV accompanied by ICAM-1 enriched microvilli projections. (5) DC actively crawl inside the LV.

The ligands of the CCR7 receptor are CCL21 and CCL19 chemokines. CCL21 is mainly produced by LEC in peripheral tissues and it is adsorbed onto heparan sulfate residues present in the ECM through its positive charged C-terminal end (75, 76). A single aminoacid variant of CCL21 (CCL21-Ser) is expressed by high endothelial venules (HEVs) and fibroblast reticular cells (FRCs) present in secondary lymphoid organs to guide T lymphocytes and DC into the T zone. CCL21 is better sensed by CCR7^+^ leukocytes when it is adsorbed onto surfaces and enhances cell mobility by a phenomenon called haptotaxis (77, 78). Besides, CCL21 can be proteolyzed by activated DC generating soluble gradients that attract DC by chemotaxis as well. Following inflammation, CCL21 biosynthesis by LEC is up-regulated and accounts for the enhanced leukocyte chemotaxis toward the LVs observed in animal models (30, 31, 79). In fact, *in vivo* confocal-based evidences show how DCs directly interact with the CCL21 patches deposited on the areas where lymphatic endothelium is deprived of basal membrane (34). From these points of entry DC subsequently crawl inside LVs (Figure 2). Interestingly, it has been demonstrated how CCL21 is needed for integrin dependent transmigration across LEC and promotes DC integrin activation through the erection from their low affinity bent conformation to the extended high affinity form (31, 80, 81). As expected, DCs lacking CCR7 do not dock successfully to CCL21 depots on LVs.

CCL19/MIP3β is a soluble CC chemokine abundantly expressed in the thymus and in FRC from the LNs but it is not expressed by LVs. Contrarily to what happens with CCL21, CCL19 lacks a negatively charged C-terminus and therefore does not bind to the glycosaminoglycans present in the ECM. In tissue, CCL19 is mainly produced by activated DCs and diffuses as soluble gradients for subsequent DC, and maybe T cells, to follow behind (82). All in all, the conjunctive activity of CCL21 and CCL19 cytokines, accounts for all CCR7-dependent DC migration toward LVs (77). Still, the specific role of each one of these chemokines in DC migration is still under discussion, although the majority of the experimental evidence shows that CCL21 is the predominant chemokine in the guidance of DC migration and intravasation (34, 78).

An interesting and unresolved paradox is that while CCR7 expression is the trademark of central memory T lymphocytes (83, 84) it is not expressed by T effector memory counterparts. The afferent lymph contains abundant CD4^+^ and CD8^+^ T cells which are CCR7^+^ as well (40, 84). It is then conceivably that some of the T cells infiltrating peripheral tissue regain CCR7 expression to enter afferent LVs in a CCR7-dependent fashion (40, 41). It has been reported that egress of CD4^+^ T cells is more efficient than CD8^+^ T cells or B cells (41) but the underlying mechanism has not been uncovered yet. To complicate things further, it has been described how under chronic inflammation dependency for outmigration on CCR7 is not total and other mechanisms are involved including random migration (42).

CXCR4 is another member of the chemokine receptor family up-regulated on DC upon maturation (74). The implications of its cognate chemokine CXCL12 in DC migration have been demonstrated *in vivo*. It has been reported that hypoxia and inflammation drives up-regulation of this chemokine in LEC (30). CXCR4 inhibition impairs DC migration in response to FITC and CHS (85). Interestingly, CXCL12 has been proposed to enhance survival and promote the maturation of DC (86).

The lipid Sphingosine-1-Phosphate (S1P) and its receptors (87) have been extensively studied in lymphocyte egress from the LN into lymph and blood (88, 89). Besides, the LVs had been reported to express Sphingosine Kinases and secrete S1P.

Although S1P expression is dispensable for homeostatic migration of DCs (90), S1P readily augments upon inflammation and presents a leading role in guiding T lymphocytes into LVs during acute inflammation (54) but is less important for memory/effector T cell egress from tissues during chronic inflammation (42). In fact, a number of reports show how S1P directs the migration of bone marrow-derived mature DC (91), skin DCs (92), or other DC subsets (93). Treatment of DCs with FTY720, a potent S1P analog that induces internalization of S1P receptors (94), block DC migration into LVs and their arrival to LNs (91). In addition, it has been reported how lack of CD69, that sequestrates S1PRs in lymphocytes (95, 96), enhances the effects of S1P-driven migration on T cell and DCs (97). Other inflammatory lipids such as PGD_2_ also sensitize memory T cells for egress from tissue to LN in a CCR7-dependent manner (98).

Recently the chemokine CX3CL1/Fractalkine has been involved in DC transit across LVs (99). This cytokine is only expressed upon inflammation and is differently processed in blood and LVs. In blood capillaries CX3CL1 is expressed predominantly as a transmembrane endothelial cytokine with both adhesive and chemoattractanct functions while in lymphatic capillaries it is, almost in its totality, shed from the basolateral surface of the lymphatic capillary and released as soluble chemokine. Importantly CX3CL1 lacks acidic residues and hence diffuses freely through the ECM promoting chemotaxis but not haptotactic migration. In fact, it seems to act as a premier inflammation-driven soluble attractor for tissue-resident DC before CCL21 is deposited on the ECM. In addition, CX3CL1 receptor (CX3CR1) impairment partially inhibits DC migration to LN and concomitant blockade of CCL21 does not show additive effects. The study of the specific mechanisms that this chemokine promotes in an *in vivo* setting deserves further research.

Another molecule involved in chemokine-driven DC migration is the chemokine decoy receptor D6. This protein is a non-signaling scavenging receptor (100, 101) that binds some inflammatory chemokines but does not bind CCL21 (102, 103). D6 is expressed on LEC surface after being cultured in the presence of inflammatory cytokines such as IL-6 or IFNγ (104). *In vitro* D6 elimination from primary LECs selectively increases the adhesion of immature DCs but not mature DC (104) and mice knocked-down for D6 showed impaired DC migration to LNs by means of macrophage accumulation around LVs (105). Therefore, it seems that D6 acts on LVs as scavenger receptor to avoid the adhesion of DC or other leukocytes that fail to express CCR7.

In our group we have been interested in the role of inflammation induced receptors in the modulation of DC transit across LVs. We have studied the participation of CD137/TNFR9/4-1BB, a receptor of the TNFR family receptors in this process. We demonstrated CD137 up-regulation in inflamed LVs. Besides, CD137 cross-linking with an agonistic mAb resulted in the up-regulation of VCAM, and increased production of CCL21 and DC accumulation close to LVs (81).

### Adhesion molecules involved in leukocyte trafficking into LVs

To traverse LVs, leukocytes must find the gaps opened in their basal membrane and situate on top of the open endothelial flaps devoid of intercellular junctions. From there, cells must squeeze their cytoplasm by cytoskeletal contraction and nuclear deformation, a process in which Rho associated protein kinases have a said (106). This transit although originally described to occur in an integrin-independent manner, is now seen as a dynamic process modulated by context dependent-factors. In fact, the participation of adhesion molecules as expressed on LVs during leukocyte transit, particularly those belonging to the family of integrin receptors, is a field under intensive investigation (Table 1). Besides, a number of receptors not belonging to CAMs family have recently been described in this process such as podoplanin, CLEVER-1, Plexin A, and CD137.

The role of ICAM-1 and VCAM in leukocyte intravasation into LVs has been controversial. Seminal studies showed a role of ICAM-1 and β2 integrins in DC migration toward LNs (107, 108). However, as mentioned above Lämmerman and colleagues showed the entrance of DCs genetically devoid of every known integrin into LVs in *ex vivo* explanted non-inflammed mouse ears (61). In contrast, TNFα, or TLR agonists induce the integrin ligands ICAM-1 and VCAM expression on LEC (29, 30, 109). These same ligands are expressed at low levels in non-inflamed LVs. Besides, blocking these CAMs or their integrin ligands inhibited the migration of DC to LNs under inflammation as well as *in vitro* assessed trans-endothelial migration (TEM) (29, 31, 53). These apparently opposing results are reconciled in light of the differential distribution of the adhesive structure under inflammatory conditions: McDonald and co-workers showed the transformation of button-like junction structures into zipper-like junctions after chronic inflammation of lung lymphatic capillaries. Thus, it seems reasonable to speculate that under these circumstances the LVs become less permeable to cell transit as a mean to regulate leukocyte transit during the resolution of tissue inflammation. In this regard, we have recently observed that ICAM-1 and VCAM integrin ligands usher DC crawling over LEC and TEM by forming microvilli like projections similar to those previously described in vascular endothelium (56, 110, 111). Such structures were only observed under inflammatory conditions and were not formed if β1 and β2 integrins were blocked on the leukocyte surface or when the CCL21 chemokine was sequestered by neutralizing monoclonal antibodies. There are no experimental data providing a direct involvement of CCL21 in the activation of β2 integrins on DC adhering to LVs *in vivo* but all the indirect evidences point in this direction. Interestingly, it has also been described how DC crawling within initial lymphatics depends on ICAM-1 only under inflammatory conditions (55). Of note, no role has been reported yet for ICAM-2 and ICAM-3 on LEC.

PECAM (CD31) is a molecule expressed on most endothelial cells and involved in leukocyte extra- and intravasation (112). LVs express less CD31 (113) than their blood counterparts and it is mostly distributed at cell–cell homotypic interactions (17, 29). Studies made with human cells have shown that blocking this molecule as well as CD99 in CXCL12 treated LEC was able to reduce TEM, both *in vitro* and on *ex vivo* tissue cultures (62). PECAM binds to integrin αvβ3 and αvβ5 integrins expressed on LVs surface, but care should be taken since PECAM is also expressed by most leukocyte subsets and might mediate homophilic interactions.

L1CAM and ALCAM have been reported to participate in leukocyte transit across LVs, although the experimental evidence provided in this regard is limited. L1CAM is a transmembrane protein widely described in neurons (114). It is also expressed in skin LC and bone marrow-derived DCs (115). L1CAM has been detected on inflamed LVs (52). This integrin ligand mediates both homophilic binding (116) and heterophilic interactions with a number of integrins (i.e., β3 and β5) (117). Recent studies described L1CAM as a protein that mediates DC adhesion and TEM across EC (52), although the molecular mechanisms involved and the relevance of this adhesion molecule are far from being clear.

ALCAM receptor mediates homophilic (ALCAM-ALCAM) or heterophilic (ALCAM-CD6) intercellular adhesion. This receptor is well established as one of the protagonist of leukocyte extravasation across blood vessels, the stabilization of the immunological synapse, and T cell activation (118) Although ALCAM function seems to be of great importance in LV organogenesis, its participation in DC migration has been described *in vitro*. However the definitive role of DC migration through LVs in lungs was not definitely proved (119).

Other adhesion molecules that do not belong to the family of integrin receptors have been related to DC adhesion to LVs. One of such examples is the LV marker podoplanin. Podoplanin has been described to bind CCL21on LEC surface with high affinity (120), and this interaction has interesting implications for lymphocyte trafficking (121, 122). Recent reports demonstrate how podoplanin expressed on LVs surface sustains DC migration and intravasation via the engagement between lectin CLEC-2 as expressed by DC and podoplanin expressed on LVs. CLEC-2 deficiency in DCs impaired their entry into lymphatics and trafficking to and within LN, thereby reducing T cell priming. Besides, the activation of CLEC-2 by podoplanin induced Rho A-mediated rearrangements of DCs actin cytoskeleton to promote motility along stromal surfaces (123). This finding is of particular interest because podoplanin is also expressed on other stromal cells and may sustain DC migration in the tissue as well as on or across LVs.

Semaphorins and their receptors, plexins and neuropilins, have been for long known as modulators of normal and pathological angiogenesis and lymphangiogenesis (124). Interestingly, the plexin-A1/sema3A axis has also been described to participate in the migration of DCs to LN. Thus, binding of sema3A to its receptor, the complex formed by plexin-A1 and neuropilin-1 expressed on the surface of the LVs, promotes DC contraction of its actomyosin cytoskeleton and squeezing across small gaps (portals) opened on the lymphatic walls (125).

Other receptors such as CLEVER-1 and the mannose receptor have been described to intervene in leukocyte transit across LVs, but their protagonism in this process is far from being clear. CLEVER-1 is a scavenger receptor expressed on LVs (126) that has also been involved in trafficking of adoptively transferred T lymphocytes from the skin to LNs (127). The Mannose receptor is a C-type lectin carbohydrate binding protein primarily present on the surface of macrophages and DCs that mediates endocytosis (128). The expression of the mannose receptor has also been described on LEC of both afferent and efferent LVs and evidence of impaired migration to LNs of DC lacking its expression has been published (113, 129). Still, its importance *in vivo* has only been shown in lymphatic sinuses inside LNs, where the absence of MR impairs lymphocyte adhesion (130).

## The Transient Synapses of LEC and Transmigrating Leukocytes: Exchange of Information to Shape the Immune Response

Beyond the control of leukocyte traffic to LNs, LECs have demonstrated to show interesting immunomodulatory functions both in afferent LVs and Lymphatic sinuses inside LNs. in these sinuses, LEC constitutively express MHC class II molecules (131) and some co-stimulatory molecules such as ICAM-1 and CD58, but do not promote allogenic T-cell proliferation (132).

In contrast, LEC of lymph node sinuses directly promote peripheral tolerance by antigen presentation to CD8 T cells (133, 134). In this work it was demonstrated that LECs are significant albeit suboptimal APC and promote peripheral tolerance by their lack from key co-stimulatory molecules such as CD80 or 4-1BBL (135) and the rapid up-regulation of significant expression levels of the co-inhibitory molecule PD-L1 (B7-H1) (134). In fact, the exploitation of the ability of LN sinuses to induce tolerance has been recently shown as an escape mechanism for B16 melanoma grafted tumors. Interestingly, the blockade of lymphangiogenic cytokine VEGF-C in this model was able to reduce LEC-induced CD8 T cell tolerance (136). The tolerogenic potentials of LEC in LN sinuses have been recently revised into detail (137).

The possibility of this immunosuppressive/tolerogenic process taking place in peripheral afferent LVs has not been investigated yet. Although IFNγ promotes MHC class II expression on dermal LEC without concomitant up-regulation of co-stimulatory molecules (unpublished observations) it is not known whether peripheral LVs can promote tolerance in such conditions. The fact that CD4^+^ T lymphocytes traffic frequently via lymphatics may set up a possibility of peripheral antigen presentation on LVs, upon CD4 T cell/LEC contact with potential consequences for instance in transplanted organs. It remains to be demonstrated whether LEC serves as a key professional antigen presenting cell for tolerance. Suggestive data on the likeliness of these phenomena has been reported in allogenic transplantation settings, where lymphocyte nodular infiltrations that resemble a tertiary lymphoid organs have been described in grafted organs, around CCL21-podoplanin complexes expressed by the LVs (120). Even more, recent findings demonstrated by intravital imaging approaches how DC crawl rather than roll once inside lymphatic capillaries (34, 55) thus favoring extensive and durable contacts between DC and LEC that may be also true for CD4 T cells and LEC. As early mentioned, tight contacts between CD4 T cells or DC and LEC are formed precisely on ICAM-1 enriched microvilli projections (56) that may facilitate a sort of “immune synapse” between leukocytes and LVs. It should not be forgotten that LFA-1 itself is an important co-stimulatory molecule for T cells (138).

In this line, immunomodulatory roles of LEC over DC have already been demonstrated. It has been reported that inflammation induced ICAM-1 is able to decrease the co-stimulation capabilities in immature and TNF-α matured (but not LPS matured) DC. This phenomenon seems to involve Mac-1 integrin ligation on DC (139). These findings raise an intriguing issue since contacts between LEC and DC or T cells do occur both under inflammation and steady–state conditions, it seems that the set of molecules that mediate such intercellular interaction are peculiar for each condition (55, 56) and most probably trigger different phenotypes in the LEC-interacting leukocyte subpopulation. For instance, the integrin ligand ICAM-1 is only engaged under inflammation. In this line, we have observed strong phospho-Tyr staining in the areas of contact between LEC Microvilli and DC (unpublished results) supporting that ICAM-1 in this specific context facilitates bidirectional crosstalk between both cell types that tiggers intercellular signals.

Another extracellular receptor involved in LEC-shaping of the immune response is CD137/4-1BB molecule. As already mentioned, we have recently identified CD137 (4-1BB) expression on inflamed LVs. The ligation of this molecule on LEC promotes CCL21 up-regulation (81) and increased DC transmigration. CD137-Ligand reverse signaling would in turn promote the increased expression of co-stimulatory molecules and chemokine receptors on migrating APCs (140). This system may be a first example of molecules denoting inflammation and subsequently fine-tuning lymphocyte activation and migration via afferent LVs. It is tempting to speculate that transit of an activated leukocyte sensitizes for subsequent transit events by a variety of immunologically relevant ligand receptor pairs, including other members of the TNF and TNFR families.

## Opportunities for Therapeutic Intervention at the Interface between LEC and Immune System Cells

### Enhancing leukocyte migration to increase vaccine efficiency

Dendritic cell based vaccines often given subcutaneously constitute an interesting approach for the treatment of cancer (141). Immunization is attempted toward defined antigens shared by tumors or against individual neoantigens product of the altered genome of individual malignancies. The latter is very attractive and encompasses strategies varying from DC loading with mRNA, tumor lysates to intratumoral DC injections.

The intradermal route of administration seems to be more effective in eliciting immune responses (142), while one of the key limitations for its efficacy seems to be DC arrival to LNs (143, 144). Since intravasation in LVs is a key step in the migration of DCs to LN, intervention in this particular step may provide increased efficiency in DC therapies. It has been reported that preconditioning the area of injection by promoting for example, acute inflammation promotes migration and maturation on DC vaccination when TLR agonist poly I:C is used as an adjuvant (145, 146). The TLR7 agonist imiquimod has also been applied to the skin at the vaccination site with a similar purpose (147). Pro-inflammatory cytokines have proved to be good local adjuvants (148).

Other methods may provide also good preconditioning for injection sites, as local irradiation (149) or laser illumination (150) which additionally enhances migratory possibilities by increasing the degradation of the basal membrane of the vessels. The preparation of DC is also particularly relevant in the migration and efficacy of DC vaccines (141). By including different cytokines in DC maturation cocktails, their migration to LNs can also be enhanced. For instance IFN-alpha (53, 151) induces increments in the expression of chemokine receptors one LEC and integrin activation on DC surface. The strongest stimulus for CCR7 expression and functionality is the lipid mediator PGE2 (152, 153). However, many immunosuppressive functions of PGE2 on DC discourage the use of this prostaglandin in DC maturation cocktails.

### The role of lymphatics on inflammatory diseases

In the last few years it has been shown how the plasticity of LV in response to inflammation can also contribute to the progression of diseases whose mechanisms involve chronic inflammation, as some autoimmune diseases (154).

Increased LV density has been observed in psoriatic skin (155) and rheumatoid arthritis lesions in mice joints (156). As previously mentioned, there is increased LV presence in kidney transplants and their secretion of CCL21 promotes DC migration and alloantigen response and rejection (120). Diminishing (157) or normalizing lymphangiogenesis by VEGFR stimulation (27) is considered a promising treatment to control chronic inflammation.

There is already some evidence for pharmacological treatments directed toward leukocyte intravasation on LVs that may help in the treatment of these diseases. For instance, the blockade of DC traffic with anti-VEGF antibodies reduces inflammation (158) and rejection of mice corneas. Interestingly, in a heart transplantation model, blockade of VEGF with antibodies has proved to decrease inflammation by a mechanism directly dependent on CCL21 production by LVs (159).

While the role of blood vessels in leukocyte traffic is known in great molecular detail, that of LV is less well understood. Regulation of adhesiveness and chemotaxis in blood vessels can be interfered for the sake of suppressing inflammation in multiple sclerosis or transplantation with anti-VLA-4 mAb. In fact, it is quite possible that the S1P antagonist sphingolimod exerts an important effect on memory lymphocyte egress from the inflamed territory suffering autoimmunity toward LN.

In our view there is also much potential in pharmacological manipulation of the CCR7 axis (160). From a drug development perspective is not an easy target but it certainly would provide a tool to disorient recirculation of pathogenic T lymphocytes and limit the arrival of immunogenic autoantigens to lymphoid tissue.

## Concluding Remarks

Research performed mainly in the last 10 years regarding leukocyte migration via afferent LVs has unraveled that the process is highly regulated and more complex than originally expected. Importantly, new incisive experimental procedures including *in vivo* imaging have provided detailed knowledge of the process. Some interesting and unexpected molecular players including chemokines and adhesion molecules have been identified as gatekeepers of LV intravasation and intriguing data about a tight relationship between LEC and leukocytes have been reported. Knowledge on the regulation of DC migration out of peripheral tissue is beginning to be exploited for vaccination, but we are only starting to learn the pathological and therapeutic implications that leukocyte-LVs contact may have. Especially, the field of the tissue-egressing mechanisms of T Lymphocytes remains neglected. We believe that a more in depth knowledge of these leukocyte-LVs interactions may provide interesting cues or potential targets for chronic inflammation. Migration through LVs must not be observed as a passive drain but as process that is highly regulated by changes in the tissue homeostasis, and that may help to shape immune responses both under steady-state and inflammatory conditions.

## Conflict of Interest Statement

Ignacio Melero has served as a consultant for Bristol Myers Squibb, Astra-Zeneca, Pfizer, Miltenyi Biotec, and Merk-Serono. The other co-authors declare that the research was conducted in the absence of any commercial or financial relationships that could be construed as a potential conflict of interest.

## References

[1] Pepper MS Skobe M Lymphatic endothelium: morphological, molecular and functional properties. J Cell Biol (2003) 163:209–13 10.1083/jcb.200308082 14581448 PMC2173536

[2] Forster R Braun A Worbs T Lymph node homing of T cells and dendritic cells via afferent lymphatics. Trends Immunol (2012) 33:271–80 10.1016/j.it.2012.02.007 22459312

[3] Mackay CR Marston WL Dudler L Naive and memory T cells show distinct pathways of lymphocyte recirculation. J Exp Med (1990) 171:801–17 10.1084/jem.171.3.801 2307933 PMC2187792

[4] Young AJ The physiology of lymphocyte migration through the single lymph node in vivo. Semin Immunol (1999) 11:73–83 10.1006/smim.1999.0163 10329494

[5] Tomura M Honda T Tanizaki H Otsuka A Egawa G Tokura Y Activated regulatory T cells are the major T cell type emigrating from the skin during a cutaneous immune response in mice. J Clin Invest (2010) 120:883–93 10.1172/JCI40926 20179354 PMC2827959

[6] Drexhage HA Mullink H De Groot J Clarke J Balfour BM A study of cells present in peripheral lymph of pigs with special reference to a type of cell resembling the Langerhans cell. Cell Tissue Res (1979) 202:407–30 10.1007/BF00220434 519712

[7] Spry CJ Pflug AJ Janossy G Humphrey JH Large mononuclear (veiled) cells like ‘Ia-like’ membrane antigens in human afferent lympn. Clin Exp Immunol (1980) 39:750–5 6991175 PMC1538145

[8] Pugh CW Macpherson GG Steer HW Characterization of nonlymphoid cells derived from rat peripheral lymph. J Exp Med (1983) 157:1758–79 10.1084/jem.157.6.1758 6854208 PMC2187049

[9] Mayrhofer G Holt PG Papadimitriou JM Functional characteristics of the veiled cells in afferent lymph from the rat intestine. Immunology (1986) 58:379–87 3525397 PMC1453468

[10] Heath WR Carbone FR Dendritic cell subsets in primary and secondary T cell responses at body surfaces. Nat Immunol (2009) 10:1237–44 10.1038/ni.1822 19915624

[11] Ohl L Mohaupt M Czeloth N Hintzen G Kiafard Z Zwirner J CCR7 governs skin dendritic cell migration under inflammatory and steady-state conditions. Immunity (2004) 21:279–88 10.1016/j.immuni.2004.06.014 15308107

[12] Scheinecker C Mchugh R Shevach EM Germain RN Constitutive presentation of a natural tissue autoantigen exclusively by dendritic cells in the draining lymph node. J Exp Med (2002) 196:1079–90 10.1084/jem.20020991 12391019 PMC2194046

[13] Mayerova D Parke EA Bursch LS Odumade OA Hogquist KA Langerhans cells activate naive self-antigen-specific CD8 T cells in the steady state. Immunity (2004) 21:391–400 10.1016/j.immuni.2004.07.019 15357950

[14] Ginhoux F Collin MP Bogunovic M Abel M Leboeuf M Helft J Blood-derived dermal Langerin+ dendritic cells survey the skin in the steady state. J Exp Med (2007) 204:3133–46 10.1084/jem.20071733 18086862 PMC2150983

[15] Wendland M Willenzon S Kocks J Davalos-Misslitz AC Hammerschmidt SI Schumann K Lymph node T cell homeostasis relies on steady state homing of dendritic cells. Immunity (2011) 35:945–57 10.1016/j.immuni.2011.10.017 22195748

[16] Kripke ML Munn CG Jeevan A Tang JM Bucana C Evidence that cutaneous antigen-presenting cells migrate to regional lymph nodes during contact sensitization. J Immunol (1990) 145:2833–8 2212665

[17] Baluk P Fuxe J Hashizume H Romano T Lashnits E Butz S Functionally specialized junctions between endothelial cells of lymphatic vessels. J Exp Med (2007) 204:2349–62 10.1084/jem.20062596 17846148 PMC2118470

[18] Casley-Smith JR Florey HW The structure of normal small lymphatics. Q J Exp Physiol Cogn Med Sci (1961) 46:101–6 13691301 10.1113/expphysiol.1961.sp001502

[19] Castenholz A Functional microanatomy of initial lymphatics with special consideration of the extracellular matrix. Lymphology (1998) 31:101–18 9793921

[20] Pflicke H Sixt M Preformed portals facilitate dendritic cell entry into afferent lymphatic vessels. J Exp Med (2009) 206:2925–35 10.1084/jem.20091739 19995949 PMC2806476

[21] Yao LC Baluk P Srinivasan RS Oliver G Mcdonald DM Plasticity of button-like junctions in the endothelium of airway lymphatics in development and inflammation. Am J Pathol (2012) 180:2561–75 10.1016/j.ajpath.2012.02.019 22538088 PMC3378913

[22] Ristimaki A Narko K Enholm B Joukov V Alitalo K Proinflammatory cytokines regulate expression of the lymphatic endothelial mitogen vascular endothelial growth factor-C. J Biol Chem (1998) 273:8413–8 10.1074/jbc.273.14.8413 9525952

[23] Kang S Lee SP Kim KE Kim HZ Memet S Koh GY Toll-like receptor 4 in lymphatic endothelial cells contributes to LPS-induced lymphangiogenesis by chemotactic recruitment of macrophages. Blood (2009) 113:2605–13 10.1182/blood-2008-07-166934 19098273

[24] Makinen T Jussila L Veikkola T Karpanen T Kettunen MI Pulkkanen KJ Inhibition of lymphangiogenesis with resulting lymphedema in transgenic mice expressing soluble VEGF receptor-3. Nat Med (2001) 7:199–205 10.1038/84651 11175851

[25] Kajiya K Sawane M Huggenberger R Detmar M Activation of the VEGFR-3 pathway by VEGF-C attenuates UVB-induced edema formation and skin inflammation by promoting lymphangiogenesis. J Invest Dermatol (2009) 129:1292–8 10.1038/jid.2008.351 19005491

[26] Flister MJ Wilber A Hall KL Iwata C Miyazono K Nisato RE Inflammation induces lymphangiogenesis through up-regulation of VEGFR-3 mediated by NF-kappaB and Prox1. Blood (2010) 115:418–29 10.1182/blood-2008-12-196840 19901262 PMC2808162

[27] Huggenberger R Ullmann S Proulx ST Pytowski B Alitalo K Detmar M Stimulation of lymphangiogenesis via VEGFR-3 inhibits chronic skin inflammation. J Exp Med (2010) 207:2255–69 10.1084/jem.20100559 20837699 PMC2947063

[28] Angeli V Randolph GJ Inflammation, lymphatic function, and dendritic cell migration. Lymphat Res Biol (2006) 4:217–28 10.1089/lrb.2006.4406 17394405

[29] Johnson LA Clasper S Holt AP Lalor PF Baban D Jackson DG An inflammation-induced mechanism for leukocyte transmigration across lymphatic vessel endothelium. J Exp Med (2006) 203:2763–77 10.1084/jem.20051759 17116732 PMC2118156

[30] Vigl B Aebischer D Nitschke M Iolyeva M Rothlin T Antsiferova O Tissue inflammation modulates gene expression of lymphatic endothelial cells and dendritic cell migration in a stimulus-dependent manner. Blood (2011) 118:205–15 10.1182/blood-2010-12-326447 21596851

[31] Johnson LA Jackson DG Inflammation-induced secretion of CCL21 in lymphatic endothelium is a key regulator of integrin-mediated dendritic cell transmigration. Int Immunol (2010) 22:839–49 10.1093/intimm/dxq435 20739459

[32] Zampell JC Avraham T Yoder N Fort N Yan A Weitman ES Lymphatic function is regulated by a coordinated expression of lymphangiogenic and anti-lymphangiogenic cytokines. Am J Physiol Cell Physiol (2012) 302:C392–404 10.1152/ajpcell.00306.2011 21940662 PMC3328842

[33] Miteva DO Rutkowski JM Dixon JB Kilarski W Shields JD Swartz MA Transmural flow modulates cell and fluid transport functions of lymphatic endothelium. Circ Res (2010) 106:920–31 10.1161/CIRCRESAHA.109.207274 20133901 PMC10994404

[34] Tal O Lim HY Gurevich I Milo I Shipony Z Ng LG DC mobilization from the skin requires docking to immobilized CCL21 on lymphatic endothelium and intralymphatic crawling. J Exp Med (2011) 208:2141–53 10.1084/jem.20102392 21930767 PMC3182054

[35] Alon R Dustin ML Force as a facilitator of integrin conformational changes during leukocyte arrest on blood vessels and antigen-presenting cells. Immunity (2007) 26:17–27 10.1016/j.immuni.2007.01.002 17241958

[36] Kunder CA St John AL Li G Leong KW Berwin B Staats HF Mast cell-derived particles deliver peripheral signals to remote lymph nodes. J Exp Med (2009) 206:2455–67 10.1084/jem.20090805 19808250 PMC2768851

[37] Kunder CA St John AL Abraham SN Mast cell modulation of the vascular and lymphatic endothelium. Blood (2011) 118:5383–93 10.1182/blood-2011-07-358432 21908429 PMC3217344

[38] Angeli V Ginhoux F Llodra J Quemeneur L Frenette PS Skobe M B cell-driven lymphangiogenesis in inflamed lymph nodes enhances dendritic cell mobilization. Immunity (2006) 24:203–15 10.1016/j.immuni.2006.01.003 16473832

[39] Halin C Detmar M An unexpected connection: lymph node lymphangiogenesis and dendritic cell migration. Immunity (2006) 24:129–31 10.1016/j.immuni.2006.01.011 16473825

[40] Bromley SK Thomas SY Luster AD Chemokine receptor CCR7 guides T cell exit from peripheral tissues and entry into afferent lymphatics. Nat Immunol (2005) 6:895–901 10.1038/ni1240 16116469

[41] Debes GF Arnold CN Young AJ Krautwald S Lipp M Hay JB Chemokine receptor CCR7 required for T lymphocyte exit from peripheral tissues. Nat Immunol (2005) 6:889–94 10.1038/ni1238 16116468 PMC2144916

[42] Brown MN Fintushel SR Lee MH Jennrich S Geherin SA Hay JB Chemoattractant receptors and lymphocyte egress from extralymphoid tissue: changing requirements during the course of inflammation. J Immunol (2010) 185:4873–82 10.4049/jimmunol.1000676 20833836 PMC3327166

[43] Yang CW Strong BS Miller MJ Unanue ER Neutrophils influence the level of antigen presentation during the immune response to protein antigens in adjuvants. J Immunol (2010) 185:2927–34 10.4049/jimmunol.1001289 20679530 PMC3509756

[44] Beauvillain C Cunin P Doni A Scotet M Jaillon S Loiry ML CCR7 is involved in the migration of neutrophils to lymph nodes. Blood (2011) 117:1196–204 10.1182/blood-2009-11-254490 21051556

[45] Alfaro C Suarez N Onate C Perez-Gracia JL Martinez-Forero I Hervas-Stubbs S Dendritic cells take up and present antigens from viable and apoptotic polymorphonuclear leukocytes. PLoS One (2011) 6:e29300 10.1371/journal.pone.0029300 22206007 PMC3243708

[46] Podgrabinska S Braun P Velasco P Kloos B Pepper MS Skobe M Molecular characterization of lymphatic endothelial cells. Proc Natl Acad Sci U S A (2002) 99:16069–74 10.1073/pnas.242401399 12446836 PMC138566

[47] Lee S Choi I Hong YK Heterogeneity and plasticity of lymphatic endothelial cells. Semin Thromb Hemost (2010) 36:352–61 10.1055/s-0030-1253457 20490985 PMC3461948

[48] Ando T Jordan P Joh T Wang Y Jennings MH Houghton J Isolation and characterization of a novel mouse lymphatic endothelial cell line: SV-LEC. Lymphat Res Biol (2005) 3:105–15 10.1089/lrb.2005.3.105 16190815

[49] Sironi M Conti A Bernasconi S Fra AM Pasqualini F Nebuloni M Generation and characterization of a mouse lymphatic endothelial cell line. Cell Tissue Res (2006) 325:91–100 10.1007/s00441-006-0171-y 16534603

[50] Kang J Ramu S Lee S Aguilar B Ganesan SK Yoo J Phosphate-buffered saline-based nucleofection of primary endothelial cells. Anal Biochem (2009) 386:251–5 10.1016/j.ab.2008.12.021 19150324 PMC2677097

[51] Zheng W Tammela T Yamamoto M Anisimov A Holopainen T Kaijalainen S Notch restricts lymphatic vessel sprouting induced by vascular endothelial growth factor. Blood (2011) 118:1154–62 10.1182/blood-2010-11-317800 21566091

[52] Maddaluno L Verbrugge SE Martinoli C Matteoli G Chiavelli A Zeng Y The adhesion molecule L1 regulates transendothelial migration and trafficking of dendritic cells. J Exp Med (2009) 206:623–35 10.1084/jem.20081211 19273627 PMC2664975

[53] Rouzaut A Garasa S Teijeira A Gonzalez I Martinez-Forero I Suarez N Dendritic cells adhere to and transmigrate across lymphatic endothelium in response to IFN-alpha. Eur J Immunol (2010) 40:3054–63 10.1002/eji.201040523 21061437

[54] Ledgerwood LG Lal G Zhang N Garin A Esses SJ Ginhoux F The sphingosine 1-phosphate receptor 1 causes tissue retention by inhibiting the entry of peripheral tissue T lymphocytes into afferent lymphatics. Nat Immunol (2008) 9:42–53 10.1038/ni1534 18037890

[55] Nitschke M Aebischer D Abadier M Haener S Lucic M Vigl B Differential requirement for ROCK in dendritic cell migration within lymphatic capillaries in steady-state and inflammation. Blood (2012) 120:2249–58 10.1182/blood-2012-03-417923 22855606

[56] Teijeira A Garasa S Pelaez R Azpilikueta A Ochoa C Marre D Lymphatic endothelium forms integrin-engaging 3D structures during DC transit across inflamed lymphatic vessels. J Invest Dermatol (2013) 133:2276–85 10.1038/jid.2013.152 23528818

[57] Khuon S Liang L Dettman RW Sporn PH Wysolmerski RB Chew TL Myosin light chain kinase mediates transcellular intravasation of breast cancer cells through the underlying endothelial cells: a three-dimensional FRET study. J Cell Sci (2010) 123:431–40 10.1242/jcs.053793 20067998 PMC2816188

[58] Shin Y Han S Jeon JS Yamamoto K Zervantonakis IK Sudo R Microfluidic assay for simultaneous culture of multiple cell types on surfaces or within hydrogels. Nat Protoc (2012) 7:1247–59 10.1038/nprot.2012.051 22678430 PMC4035049

[59] Zervantonakis IK Hughes-Alford SK Charest JL Condeelis JS Gertler FB Kamm RD Three-dimensional microfluidic model for tumor cell intravasation and endothelial barrier function. Proc Natl Acad Sci U S A (2012) 109:13515–20 10.1073/pnas.1210182109 22869695 PMC3427099

[60] Larsen CP Steinman RM Witmer-Pack M Hankins DF Morris PJ Austyn JM Migration and maturation of Langerhans cells in skin transplants and explants. J Exp Med (1990) 172:1483–93 10.1084/jem.172.5.1483 2230654 PMC2188669

[61] Lammermann T Bader BL Monkley SJ Worbs T Wedlich-Soldner R Hirsch K Rapid leukocyte migration by integrin-independent flowing and squeezing. Nature (2008) 453:51–5 10.1038/nature06887 18451854

[62] Torzicky M Viznerova P Richter S Strobl H Scheinecker C Foedinger D Platelet endothelial cell adhesion molecule-1 (PECAM-1/CD31) and CD99 are critical in lymphatic transmigration of human dendritic cells. J Invest Dermatol (2012) 132:1149–57 10.1038/jid.2011.420 22189791

[63] Randolph GJ Angeli V Swartz MA Dendritic-cell trafficking to lymph nodes through lymphatic vessels. Nat Rev Immunol (2005) 5:617–28 10.1038/nri1670 16056255

[64] Thomas WR Edwards AJ Watkins MC Asherson GL Distribution of immunogenic cells after painting with the contact sensitizers fluorescein isothiocyanate and oxazolone. Different sensitizers form immunogenic complexes with different cell populations. Immunology (1980) 39:21–7 6991394 PMC1457785

[65] Randolph GJ Inaba K Robbiani DF Steinman RM Muller WA Differentiation of phagocytic monocytes into lymph node dendritic cells in vivo. Immunity (1999) 11:753–61 10.1016/S1074-7613(00)80149-1 10626897

[66] Kimber I Cumberbatch M Stimulation of Langerhans cell migration by tumor necrosis factor alpha (TNF-alpha). J Invest Dermatol (1992) 99:48S–50S 10.1111/1523-1747.ep12668986 1431209

[67] Martin-Fontecha A Sebastiani S Hopken UE Uguccioni M Lipp M Lanzavecchia A Regulation of dendritic cell migration to the draining lymph node: impact on T lymphocyte traffic and priming. J Exp Med (2003) 198:615–21 10.1084/jem.20030448 12925677 PMC2194169

[68] Choi I Chung HK Ramu S Lee HN Kim KE Lee S Visualization of lymphatic vessels by Prox1-promoter directed GFP reporter in a bacterial artificial chromosome-based transgenic mouse. Blood (2011) 117:362–5 10.1182/blood-2010-07-298562 20962325 PMC3037757

[69] Hagerling R Pollmann C Kremer L Andresen V Kiefer F Intravital two-photon microscopy of lymphatic vessel development and function using a transgenic Prox1 promoter-directed mOrange2 reporter mouse. Biochem Soc Trans (2011) 39:1674–81 10.1042/BST20110722 22103506

[70] Martinez-Corral I Olmeda D Dieguez-Hurtado R Tammela T Alitalo K Ortega S In vivo imaging of lymphatic vessels in development, wound healing, inflammation, and tumor metastasis. Proc Natl Acad Sci U S A (2012) 109:6223–8 10.1073/pnas.1115542109 22474390 PMC3341065

[71] Svingen T Francois M Wilhelm D Koopman P Three-dimensional imaging of Prox1-EGFP transgenic mouse gonads reveals divergent modes of lymphangiogenesis in the testis and ovary. PLoS One (2012) 7:e52620 10.1371/journal.pone.0052620 23285114 PMC3527586

[72] Truman LA Bentley KL Smith EC Massaro SA Gonzalez DG Haberman AM ProxTom lymphatic vessel reporter mice reveal Prox1 expression in the adrenal medulla, megakaryocytes, and platelets. Am J Pathol (2012) 180:1715–25 10.1016/j.ajpath.2011.12.026 22310467 PMC3349900

[73] Kilarski WW Guc E Teo JC Oliver SR Lund AW Swartz MA Intravital immunofluorescence for visualizing the microcirculatory and immune microenvironments in the mouse ear dermis. PLoS One (2013) 8:e57135 10.1371/journal.pone.0057135 23451163 PMC3581585

[74] Sallusto F Schaerli P Loetscher P Schaniel C Lenig D Mackay CR Rapid and coordinated switch in chemokine receptor expression during dendritic cell maturation. Eur J Immunol (1998) 28:2760–9 10.1002/(SICI)1521-4141(199809)28:09<2760::AID-IMMU2760>3.0.CO;2-N 9754563

[75] Kriehuber E Breiteneder-Geleff S Groeger M Soleiman A Schoppmann SF Stingl G Isolation and characterization of dermal lymphatic and blood endothelial cells reveal stable and functionally specialized cell lineages. J Exp Med (2001) 194:797–808 10.1084/jem.194.6.797 11560995 PMC2195953

[76] Bao X Moseman EA Saito H Petryniak B Thiriot A Hatakeyama S Endothelial heparan sulfate controls chemokine presentation in recruitment of lymphocytes and dendritic cells to lymph nodes. Immunity (2010) 33:817–29 10.1016/j.immuni.2010.10.018 21093315 PMC2996097

[77] Schumann K Lammermann T Bruckner M Legler DF Polleux J Spatz JP Immobilized chemokine fields and soluble chemokine gradients cooperatively shape migration patterns of dendritic cells. Immunity (2010) 32:703–13 10.1016/j.immuni.2010.04.017 20471289

[78] Weber M Hauschild R Schwarz J Moussion C De Vries I Legler DF Interstitial dendritic cell guidance by haptotactic chemokine gradients. Science (2013) 339:328–32 10.1126/science.1228456 23329049

[79] Martin-Fontecha A Lanzavecchia A Sallusto F Dendritic cell migration to peripheral lymph nodes. Handb Exp Pharmacol (2009) 188:31–49 10.1007/978-3-540-71029-5_2 19031020

[80] Eich C De Vries IJ Linssen PC De Boer A Boezeman JB Figdor CG The lymphoid chemokine CCL21 triggers LFA-1 adhesive properties on human dendritic cells. Immunol Cell Biol (2011) 89:458–65 10.1038/icb.2010.103 20805842

[81] Teijeira A Palazon A Garasa S Marre D Auba C Rogel A CD137 on inflamed lymphatic endothelial cells enhances CCL21-guided migration of dendritic cells. FASEB J (2012) 26:3380–92 10.1096/fj.11-201061 22593548

[82] Shields JD Fleury ME Yong C Tomei AA Randolph GJ Swartz MA Autologous chemotaxis as a mechanism of tumor cell homing to lymphatics via interstitial flow and autocrine CCR7 signaling. Cancer Cell (2007) 11:526–38 10.1016/j.ccr.2007.04.020 17560334

[83] Sallusto F Lenig D Forster R Lipp M Lanzavecchia A Two subsets of memory T lymphocytes with distinct homing potentials and effector functions. Nature (1999) 401:708–12 10.1038/44385 10537110

[84] Masopust D Schenkel JM The integration of T cell migration, differentiation and function. Nat Rev Immunol (2013) 13:309–20 10.1038/nri3442 23598650

[85] Kabashima K Shiraishi N Sugita K Mori T Onoue A Kobayashi M CXCL12-CXCR4 engagement is required for migration of cutaneous dendritic cells. Am J Pathol (2007) 171:1249–57 10.2353/ajpath.2007.070225 17823289 PMC1988874

[86] Kabashima K Sugita K Shiraishi N Tamamura H Fujii N Tokura Y CXCR4 engagement promotes dendritic cell survival and maturation. Biochem Biophys Res Commun (2007) 361:1012–6 10.1016/j.bbrc.2007.07.128 17679142

[87] Spiegel S Milstien S The outs and the ins of sphingosine-1-phosphate in immunity. Nat Rev Immunol (2011) 11:403–15 10.1038/nri2974 21546914 PMC3368251

[88] Matloubian M Lo CG Cinamon G Lesneski MJ Xu Y Brinkmann V Lymphocyte egress from thymus and peripheral lymphoid organs is dependent on S1P receptor 1. Nature (2004) 427:355–60 10.1038/nature02284 14737169

[89] Pappu R Schwab SR Cornelissen I Pereira JP Regard JB Xu Y Promotion of lymphocyte egress into blood and lymph by distinct sources of sphingosine-1-phosphate. Science (2007) 316:295–8 10.1126/science.1139221 17363629

[90] Pham TH Baluk P Xu Y Grigorova I Bankovich AJ Pappu R Lymphatic endothelial cell sphingosine kinase activity is required for lymphocyte egress and lymphatic patterning. J Exp Med (2010) 207:17–27 10.1084/jem.20091619 20026661 PMC2812554

[91] Czeloth N Bernhardt G Hofmann F Genth H Forster R Sphingosine-1-phosphate mediates migration of mature dendritic cells. J Immunol (2005) 175:2960–7 16116182 10.4049/jimmunol.175.5.2960

[92] Gollmann G Neuwirt H Tripp CH Mueller H Konwalinka G Heufler C Sphingosine-1-phosphate receptor type-1 agonism impairs blood dendritic cell chemotaxis and skin dendritic cell migration to lymph nodes under inflammatory conditions. Int Immunol (2008) 20:911–23 10.1093/intimm/dxn050 18495625

[93] Rathinasamy A Czeloth N Pabst O Forster R Bernhardt G The origin and maturity of dendritic cells determine the pattern of sphingosine 1-phosphate receptors expressed and required for efficient migration. J Immunol (2010) 185:4072–81 10.4049/jimmunol.1000568 20826749

[94] Graler MH Goetzl EJ The immunosuppressant FTY720 down-regulates sphingosine 1-phosphate G-protein-coupled receptors. FASEB J (2004) 18:551–3 10.1096/fj.03-0910fje 14715694

[95] Shiow LR Rosen DB Brdickova N Xu Y An J Lanier LL CD69 acts downstream of interferon-alpha/beta to inhibit S1P1 and lymphocyte egress from lymphoid organs. Nature (2006) 440:540–4 10.1038/nature04606 16525420

[96] Bankovich AJ Shiow LR Cyster JG CD69 suppresses sphingosine 1-phosphate receptor-1 (S1P1) function through interaction with membrane helix 4. J Biol Chem (2010) 285:22328–37 10.1074/jbc.M110.123299 20463015 PMC2903414

[97] Lamana A Martin P De La Fuente H Martinez-Munoz L Cruz-Adalia A Ramirez-Huesca M CD69 modulates sphingosine-1-phosphate-induced migration of skin dendritic cells. J Invest Dermatol (2011) 131:1503–12 10.1038/jid.2011.54 21412255

[98] Ahmed SR Mcgettrick HM Yates CM Buckley CD Ratcliffe MJ Nash GB Prostaglandin D2 regulates CD4+ memory T cell trafficking across blood vascular endothelium and primes these cells for clearance across lymphatic endothelium. J Immunol (2011) 187:1432–9 10.4049/jimmunol.1100299 21715691

[99] Johnson LA Jackson DG The chemokine CX3CL1 promotes trafficking of dendritic cells through inflamed lymphatics. J Cell Sci (2013) 126:5259–70 10.1242/jcs.135343 24006262 PMC3828594

[100] Fra AM Locati M Otero K Sironi M Signorelli P Massardi ML Cutting edge: scavenging of inflammatory CC chemokines by the promiscuous putatively silent chemokine receptor D6. J Immunol (2003) 170:2279–82 12594248 10.4049/jimmunol.170.5.2279

[101] Weber M Blair E Simpson CV O’Hara M Blackburn PE Rot A The chemokine receptor D6 constitutively traffics to and from the cell surface to internalize and degrade chemokines. Mol Biol Cell (2004) 15:2492–508 10.1091/mbc.E03-09-0634 15004236 PMC404040

[102] Bonecchi R Locati M Galliera E Vulcano M Sironi M Fra AM Differential recognition and scavenging of native and truncated macrophage-derived chemokine (macrophage-derived chemokine/CC chemokine ligand 22) by the D6 decoy receptor. J Immunol (2004) 172:4972–6 15067078 10.4049/jimmunol.172.8.4972

[103] Savino B Borroni EM Torres NM Proost P Struyf S Mortier A Recognition versus adaptive up-regulation and degradation of CC chemokines by the chemokine decoy receptor D6 are determined by their N-terminal sequence. J Biol Chem (2009) 284:26207–15 10.1074/jbc.M109.029249 19632987 PMC2758019

[104] McKimmie CS Singh MD Hewit K Lopez-Franco O Le Brocq M Rose-John S An analysis of the function and expression of D6 on lymphatic endothelial cells. Blood (2013) 121:3768–77 10.1182/blood-2012-04-425314 23479571

[105] Lee KM Mckimmie CS Gilchrist DS Pallas KJ Nibbs RJ Garside P D6 facilitates cellular migration and fluid flow to lymph nodes by suppressing lymphatic congestion. Blood (2011) 118:6220–9 10.1182/blood-2011-03-344044 21979941 PMC3234674

[106] Vicente-Manzanares M Ma X Adelstein RS Horwitz AR Non-muscle myosin II takes centre stage in cell adhesion and migration. Nat Rev Mol Cell Biol (2009) 10:778–90 10.1038/nrm2786 19851336 PMC2834236

[107] Ma J Wang JH Guo YJ Sy MS Bigby M In vivo treatment with anti-ICAM-1 and anti-LFA-1 antibodies inhibits contact sensitization-induced migration of epidermal Langerhans cells to regional lymph nodes. Cell Immunol (1994) 158:389–99 10.1006/cimm.1994.1285 7923390

[108] Xu H Guan H Zu G Bullard D Hanson J Slater M The role of ICAM-1 molecule in the migration of Langerhans cells in the skin and regional lymph node. Eur J Immunol (2001) 31:3085–93 10.1002/1521-4141(2001010)31:10<3085::AID-IMMU3085>3.0.CO;2-B 11592085 PMC4894309

[109] Pegu A Qin S Fallert Junecko BA Nisato RE Pepper MS Reinhart TA Human lymphatic endothelial cells express multiple functional TLRs. J Immunol (2008) 180:3399–405 18292566 10.4049/jimmunol.180.5.3399

[110] Barreiro O Yanez-Mo M Serrador JM Montoya MC Vicente-Manzanares M Tejedor R Dynamic interaction of VCAM-1 and ICAM-1 with moesin and ezrin in a novel endothelial docking structure for adherent leukocytes. J Cell Biol (2002) 157:1233–45 10.1083/jcb.200112126 12082081 PMC2173557

[111] Carman CV Jun CD Salas A Springer TA Endothelial cells proactively form microvilli-like membrane projections upon intercellular adhesion molecule 1 engagement of leukocyte LFA-1. J Immunol (2003) 171:6135–44 14634129 10.4049/jimmunol.171.11.6135

[112] Nourshargh S Hordijk PL Sixt M Breaching multiple barriers: leukocyte motility through venular walls and the interstitium. Nat Rev Mol Cell Biol (2010) 11:366–78 10.1038/nrm2889 20414258

[113] Hirakawa S Hong YK Harvey N Schacht V Matsuda K Libermann T Identification of vascular lineage-specific genes by transcriptional profiling of isolated blood vascular and lymphatic endothelial cells. Am J Pathol (2003) 162:575–86 10.1016/S0002-9440(10)63851-5 12547715 PMC1851142

[114] Maness PF Schachner M Neural recognition molecules of the immunoglobulin superfamily: signaling transducers of axon guidance and neuronal migration. Nat Neurosci (2007) 10:19–26 10.1038/nn0207-263b 17189949

[115] Pancook JD Reisfeld RA Varki N Vitiello A Fox RI Montgomery AM Expression and regulation of the neural cell adhesion molecule L1 on human cells of myelomonocytic and lymphoid origin. J Immunol (1997) 158:4413–21 9127006

[116] Wei CH Ryu SE Homophilic interaction of the L1 family of cell adhesion molecules. Exp Mol Med (2012) 44:413–23 10.3858/emm.2012.44.7.050 22573111 PMC3406286

[117] Felding-Habermann B Silletti S Mei F Siu CH Yip PM Brooks PC A single immunoglobulin-like domain of the human neural cell adhesion molecule L1 supports adhesion by multiple vascular and platelet integrins. J Cell Biol (1997) 139:1567–81 10.1083/jcb.139.6.1567 9396761 PMC2132622

[118] Gilsanz A Sanchez-Martin L Gutierrez-Lopez MD Ovalle S Machado-Pineda Y Reyes R ALCAM/CD166 adhesive function is regulated by the tetraspanin CD9. Cell Mol Life Sci (2013) 70:475–93 10.1007/s00018-012-1132-0 23052204 PMC11113661

[119] Iolyeva M Karaman S Willrodt AH Weingartner S Vigl B Halin C Novel role for ALCAM in lymphatic network formation and function. FASEB J (2013) 27:978–90 10.1096/fj.12-217844 23169771

[120] Kerjaschki D Regele HM Moosberger I Nagy-Bojarski K Watschinger B Soleiman A Lymphatic neoangiogenesis in human kidney transplants is associated with immunologically active lymphocytic infiltrates. J Am Soc Nephrol (2004) 15:603–12 10.1097/01.ASN.0000113316.52371.2E 14978162

[121] Luther SA Tang HL Hyman PL Farr AG Cyster JG Coexpression of the chemokines ELC and SLC by T zone stromal cells and deletion of the ELC gene in the plt/plt mouse. Proc Natl Acad Sci U S A (2000) 97:12694–9 10.1073/pnas.97.23.12694 11070085 PMC18826

[122] Bajenoff M Egen JG Koo LY Laugier JP Brau F Glaichenhaus N Stromal cell networks regulate lymphocyte entry, migration, and territoriality in lymph nodes. Immunity (2006) 25:989–1001 10.1016/j.immuni.2006.10.011 17112751 PMC2692293

[123] Acton SE Astarita JL Malhotra D Lukacs-Kornek V Franz B Hess PR Podoplanin-rich stromal networks induce dendritic cell motility via activation of the C-type lectin receptor CLEC-2. Immunity (2012) 37:276–89 10.1016/j.immuni.2012.05.022 22884313 PMC3556784

[124] Sakurai A Doci CL Gutkind JS Semaphorin signaling in angiogenesis, lymphangiogenesis and cancer. Cell Res (2012) 22:23–32 10.1038/cr.2011.198 22157652 PMC3351930

[125] Takamatsu H Takegahara N Nakagawa Y Tomura M Taniguchi M Friedel RH Semaphorins guide the entry of dendritic cells into the lymphatics by activating myosin II. Nat Immunol (2010) 11:594–600 10.1038/ni.1885 20512151 PMC3045806

[126] Salmi M Koskinen K Henttinen T Elima K Jalkanen S CLEVER-1 mediates lymphocyte transmigration through vascular and lymphatic endothelium. Blood (2004) 104:3849–57 10.1182/blood-2004-01-0222 15297319

[127] Karikoski M Irjala H Maksimow M Miiluniemi M Granfors K Hernesniemi S Clever-1/stabilin-1 regulates lymphocyte migration within lymphatics and leukocyte entrance to sites of inflammation. Eur J Immunol (2009) 39:3477–87 10.1002/eji.200939896 19830743

[128] Gazi U Martinez-Pomares L Influence of the mannose receptor in host immune responses. Immunobiology (2009) 214:554–61 10.1016/j.imbio.2008.11.004 19162368

[129] Irjala H Johansson EL Grenman R Alanen K Salmi M Jalkanen S Mannose receptor is a novel ligand for L-selectin and mediates lymphocyte binding to lymphatic endothelium. J Exp Med (2001) 194:1033–42 10.1084/jem.194.8.1033 11602634 PMC2193520

[130] Marttila-Ichihara F Turja R Miiluniemi M Karikoski M Maksimow M Niemela J Macrophage mannose receptor on lymphatics controls cell trafficking. Blood (2008) 112:64–72 10.1182/blood-2007-10-118984 18434610

[131] Amatschek S Kriehuber E Bauer W Reininger B Meraner P Wolpl A Blood and lymphatic endothelial cell-specific differentiation programs are stringently controlled by the tissue environment. Blood (2007) 109:4777–85 10.1182/blood-2006-10-053280 17289814

[132] Norder M Gutierrez MG Zicari S Cervi E Caruso A Guzman CA Lymph node-derived lymphatic endothelial cells express functional costimulatory molecules and impair dendritic cell-induced allogenic T-cell proliferation. FASEB J (2012) 26:2835–46 10.1096/fj.12-205278 22459150

[133] Cohen JN Guidi CJ Tewalt EF Qiao H Rouhani SJ Ruddell A Lymph node-resident lymphatic endothelial cells mediate peripheral tolerance via Aire-independent direct antigen presentation. J Exp Med (2010) 207:681–8 10.1084/jem.20092465 20308365 PMC2856027

[134] Tewalt EF Cohen JN Rouhani SJ Guidi CJ Qiao H Fahl SP Lymphatic endothelial cells induce tolerance via PD-L1 and lack of costimulation leading to high-level PD-1 expression on CD8 T cells. Blood (2012) 120:4772–82 10.1182/blood-2012-04-427013 22993390 PMC3520619

[135] Melero I Bach N Chen L Costimulation, tolerance and ignorance of cytolytic T lymphocytes in immune responses to tumor antigens. Life Sci (1997) 60:2035–41 10.1016/S0024-3205(96)00686-8 9180357

[136] Lund AW Duraes FV Hirosue S Raghavan VR Nembrini C Thomas SN VEGF-C promotes immune tolerance in B16 melanomas and cross-presentation of tumor antigen by lymph node lymphatics. Cell Rep (2012) 1:191–9 10.1016/j.celrep.2012.01.005 22832193

[137] Tewalt EF Cohen JN Rouhani SJ Engelhard VH Lymphatic endothelial cells – key players in regulation of tolerance and immunity. Front Immunol (2012) 3:305 10.3389/fimmu.2012.00305 23060883 PMC3460259

[138] Sims TN Dustin ML The immunological synapse: integrins take the stage. Immunol Rev (2002) 186:100–17 10.1034/j.1600-065X.2002.18610.x 12234366

[139] Podgrabinska S Kamalu O Mayer L Shimaoka M Snoeck H Randolph GJ Inflamed lymphatic endothelium suppresses dendritic cell maturation and function via Mac-1/ICAM-1-dependent mechanism. J Immunol (2009) 183:1767–79 10.4049/jimmunol.0802167 19587009 PMC4410990

[140] Lippert U Zachmann K Ferrari DM Schwarz H Brunner E Mahbub-Ul Latif AH CD137 ligand reverse signaling has multiple functions in human dendritic cells during an adaptive immune response. Eur J Immunol (2008) 38:1024–32 10.1002/eji.200737800 18395851

[141] Palucka K Banchereau J Cancer immunotherapy via dendritic cells. Nat Rev Cancer (2012) 12:265–77 10.1038/nrc3258 22437871 PMC3433802

[142] Lesterhuis WJ De Vries IJ Schreibelt G Lambeck AJ Aarntzen EH Jacobs JF Route of administration modulates the induction of dendritic cell vaccine-induced antigen-specific T cells in advanced melanoma patients. Clin Cancer Res (2011) 17:5725–35 10.1158/1078-0432.CCR-11-1261 21771874

[143] De Vries IJ Krooshoop DJ Scharenborg NM Lesterhuis WJ Diepstra JH Van Muijen GN Effective migration of antigen-pulsed dendritic cells to lymph nodes in melanoma patients is determined by their maturation state. Cancer Res (2003) 63:12–7 12517769

[144] de Vries IJ Lesterhuis WJ Barentsz JO Verdijk P Van Krieken JH Boerman OC Magnetic resonance tracking of dendritic cells in melanoma patients for monitoring of cellular therapy. Nat Biotechnol (2005) 23:1407–13 10.1038/nbt1154 16258544

[145] Nair S Mclaughlin C Weizer A Su Z Boczkowski D Dannull J Injection of immature dendritic cells into adjuvant-treated skin obviates the need for ex vivo maturation. J Immunol (2003) 171:6275–82 14634145 10.4049/jimmunol.171.11.6275

[146] Tripp CH Ebner S Ratzinger G Romani N Stoitzner P Conditioning of the injection site with CpG enhances the migration of adoptively transferred dendritic cells and endogenous CD8^+^ T-cell responses. J Immunother (2010) 33:115–25 10.1097/CJI.0b013e3181b8ef5f 20145551

[147] Prins RM Craft N Bruhn KW Khan-Farooqi H Koya RC Stripecke R The TLR-7 agonist, imiquimod, enhances dendritic cell survival and promotes tumor antigen-specific T cell priming: relation to central nervous system antitumor immunity. J Immunol (2006) 176:157–64 16365406 10.4049/jimmunol.176.1.157

[148] Bracci L La Sorsa V Belardelli F Proietti E Type I interferons as vaccine adjuvants against infectious diseases and cancer. Expert Rev Vaccines (2008) 7:373–81 10.1586/14760584.7.3.373 18393607

[149] Cummings RJ Gerber SA Judge JL Ryan JL Pentland AP Lord EM Exposure to ionizing radiation induces the migration of cutaneous dendritic cells by a CCR7-dependent mechanism. J Immunol (2012) 189:4247–57 10.4049/jimmunol.1201371 23002435 PMC3478420

[150] Chen X Zeng Q Wu MX Improved efficacy of dendritic cell-based immunotherapy by cutaneous laser illumination. Clin Cancer Res (2012) 18:2240–9 10.1158/1078-0432.CCR-11-2654 22392913 PMC3328606

[151] Alfaro C Perez-Gracia JL Suarez N Rodriguez J Fernandez De Sanmamed M Sangro B Pilot clinical trial of type 1 dendritic cells loaded with autologous tumor lysates combined with GM-CSF, pegylated IFN, and cyclophosphamide for metastatic cancer patients. J Immunol (2011) 187:6130–42 10.4049/jimmunol.1102209 22048768

[152] Scandella E Men Y Gillessen S Forster R Groettrup M Prostaglandin E2 is a key factor for CCR7 surface expression and migration of monocyte-derived dendritic cells. Blood (2002) 100:1354–61 10.1182/blood-2001-11-0017 12149218

[153] Kabashima K Sakata D Nagamachi M Miyachi Y Inaba K Narumiya S Prostaglandin E2-EP4 signaling initiates skin immune responses by promoting migration and maturation of Langerhans cells. Nat Med (2003) 9:744–9 10.1038/nm872 12740571

[154] Cueni LN Detmar M The lymphatic system in health and disease. Lymphat Res Biol (2008) 6:109–22 10.1089/lrb.2008.1008 19093783 PMC3572233

[155] Kunstfeld R Hirakawa S Hong YK Schacht V Lange-Asschenfeldt B Velasco P Induction of cutaneous delayed-type hypersensitivity reactions in VEGF-A transgenic mice results in chronic skin inflammation associated with persistent lymphatic hyperplasia. Blood (2004) 104:1048–57 10.1182/blood-2003-08-2964 15100155

[156] Zhang Q Lu Y Proulx ST Guo R Yao Z Schwarz EM Increased lymphangiogenesis in joints of mice with inflammatory arthritis. Arthritis Res Ther (2007) 9:R118 10.1186/ar2326 17997858 PMC2246237

[157] Yin N Zhang N Xu J Shi Q Ding Y Bromberg JS Targeting lymphangiogenesis after islet transplantation prolongs isletallograft survival. Transplantation (2011) 92:25–30 10.1097/TP.0b013e31821d2661 21508896 PMC3703312

[158] Chen L Hamrah P Cursiefen C Zhang Q Pytowski B Streilein JW Vascular endothelial growth factor receptor-3 mediates induction of corneal alloimmunity. Nat Med (2004) 10:813–5 10.1038/nm1078 15235599

[159] Nykanen AI Sandelin H Krebs R Keranen MA Tuuminen R Karpanen T Targeting lymphatic vessel activation and CCL21 production by vascular endothelial growth factor receptor-3 inhibition has novel immunomodulatory and antiarteriosclerotic effects in cardiac allografts. Circulation (2010) 121:1413–22 10.1161/CIRCULATIONAHA.109.910703 20231530

[160] Sasaki M Hasegawa H Kohno M Inoue A Ito MR Fujita S Antagonist of secondary lymphoid-tissue chemokine (CCR ligand 21) prevents the development of chronic graft-versus-host disease in mice. J Immunol (2003) 170:588–96 12496447 10.4049/jimmunol.170.1.588

[161] Cera MR Fabbri M Molendini C Corada M Orsenigo F Rehberg M JAM-A promotes neutrophil chemotaxis by controlling integrin internalization and recycling. J Cell Sci (2009) 122:268–77 10.1242/jcs.037127 19118219

